# Supplementary material for: A CandiChrome toolkit for multicolor labeling of Candida cells
Source: bioRxiv. 2026 May 12:2026.05.11.723596. Preprint. [Version 1] doi: 10.64898/2026.05.11.723596 (PMC13192982; doi:10.64898/2026.05.11.723596)
Supplement: Supplement 1 [file media-1.pdf]

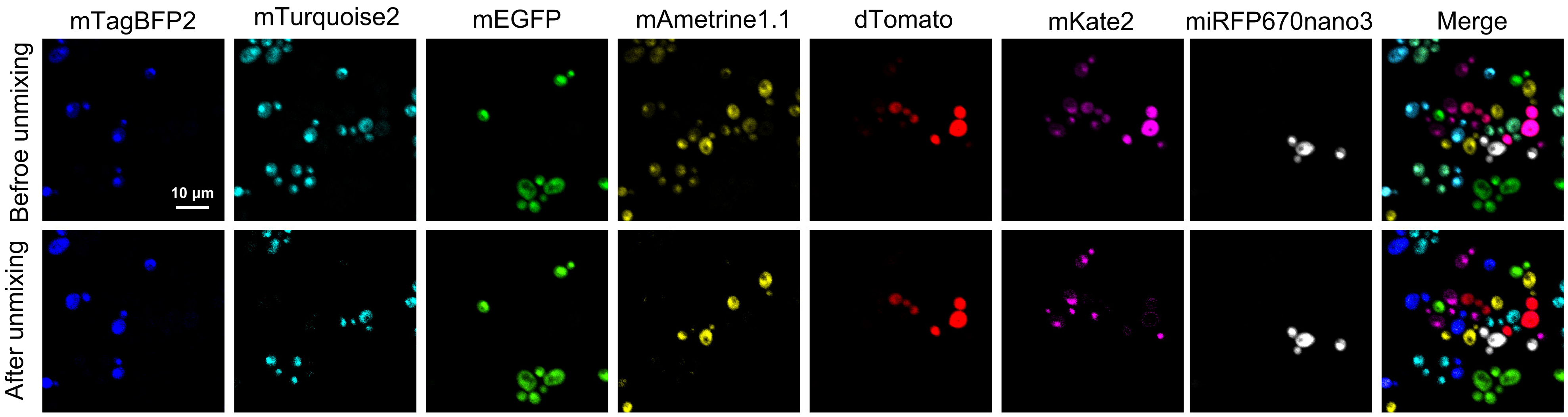

Figure S1. Spectral unmixing of the CandiChrome panel using an Evident FV3000 confocal microscope. Representative fluorescence images of mixed *C. albicans* populations expressing the indicated CandiChrome fluorophores before and after spectral unmixing. Cells were grown overnight in SCD at 30 °C and imaged on an FV3000 confocal microscope. Unmixing was performed using the built-in software. Scale bar, 10 µm.

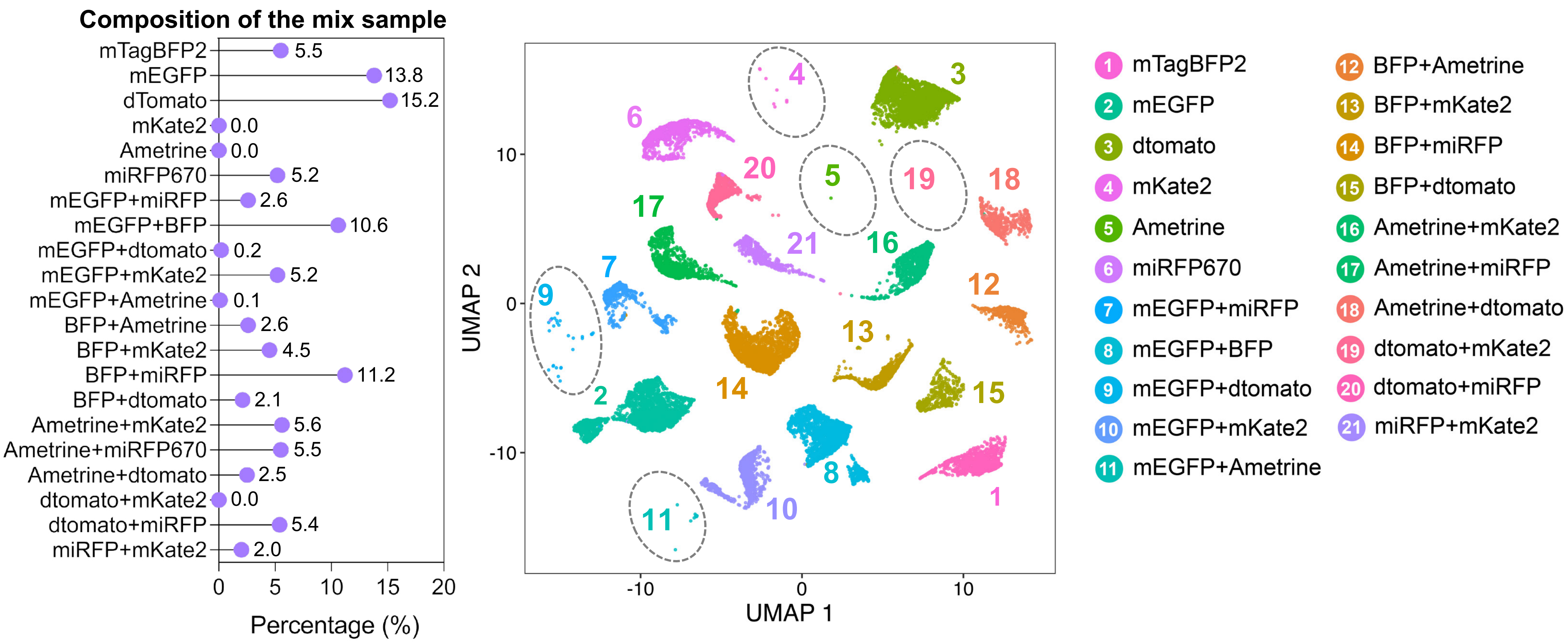

Figure S2. Resolution of a 16-population sample by flow cytometry and UMAP.
Composition of the mixed sample (left), UMAP projection (center), and population labels (right). Data were acquired on a Cytek Aurora flow cytometer and analyzed in R.

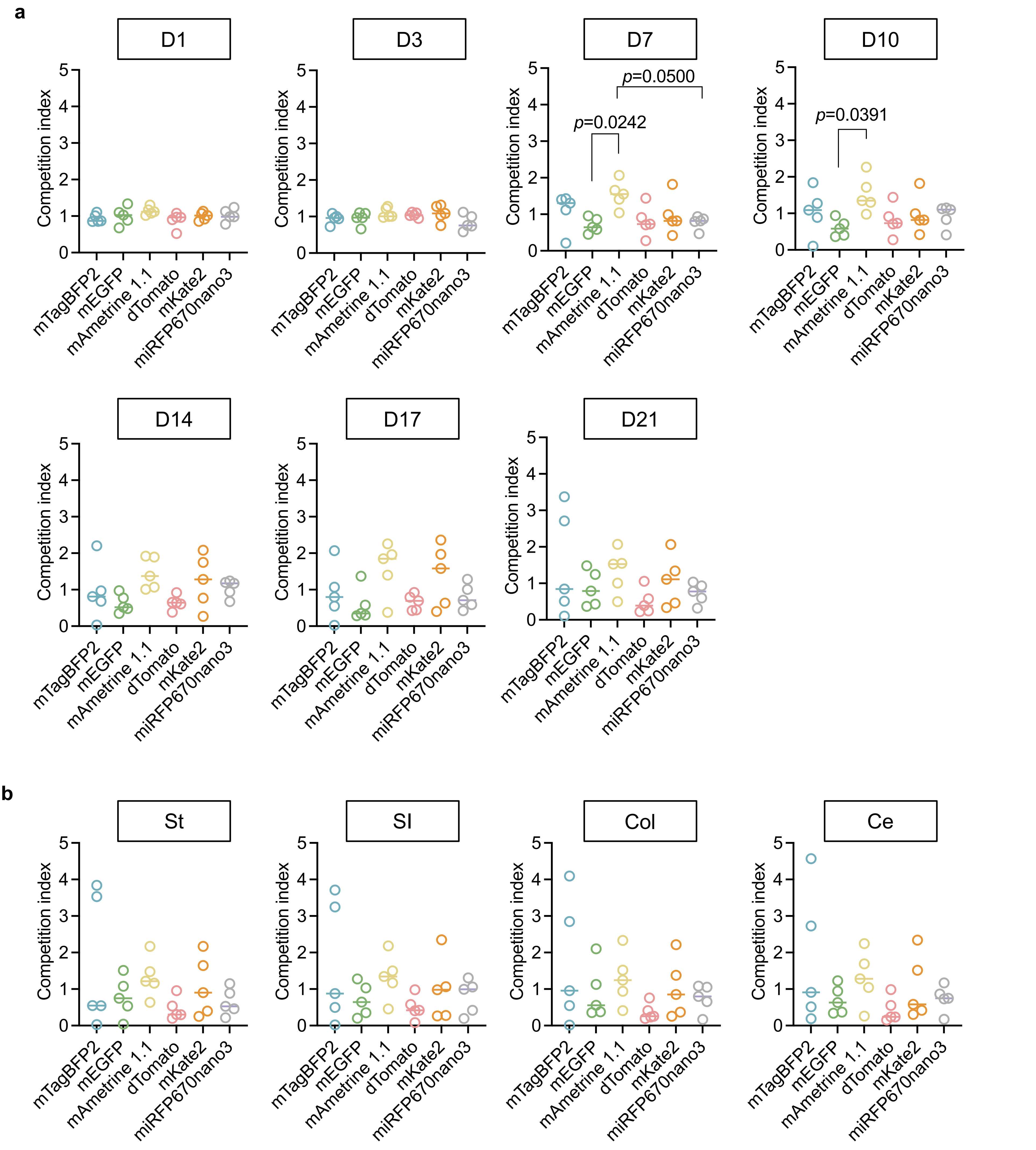

Figure S3. Competition indices of CandiChrome-labeled strains during gastrointestinal colonization.
a, Competition index values for each CandiChrome-labeled strain recovered from fecal samples at the indicated time points. Each symbol represents one mouse; horizontal lines indicate the mean. **b,** Competition indices for each strain in gastrointestinal tissues at endpoint. St, stomach; SI, small intestine; Col, colon; Ce, cecum. Each symbol represents one mouse; horizontal lines indicate the mean. Statistical significance was assessed by ordinary one-way ANOVA with Tukey's multiple-comparisons test. Exact P values are shown in the figure.

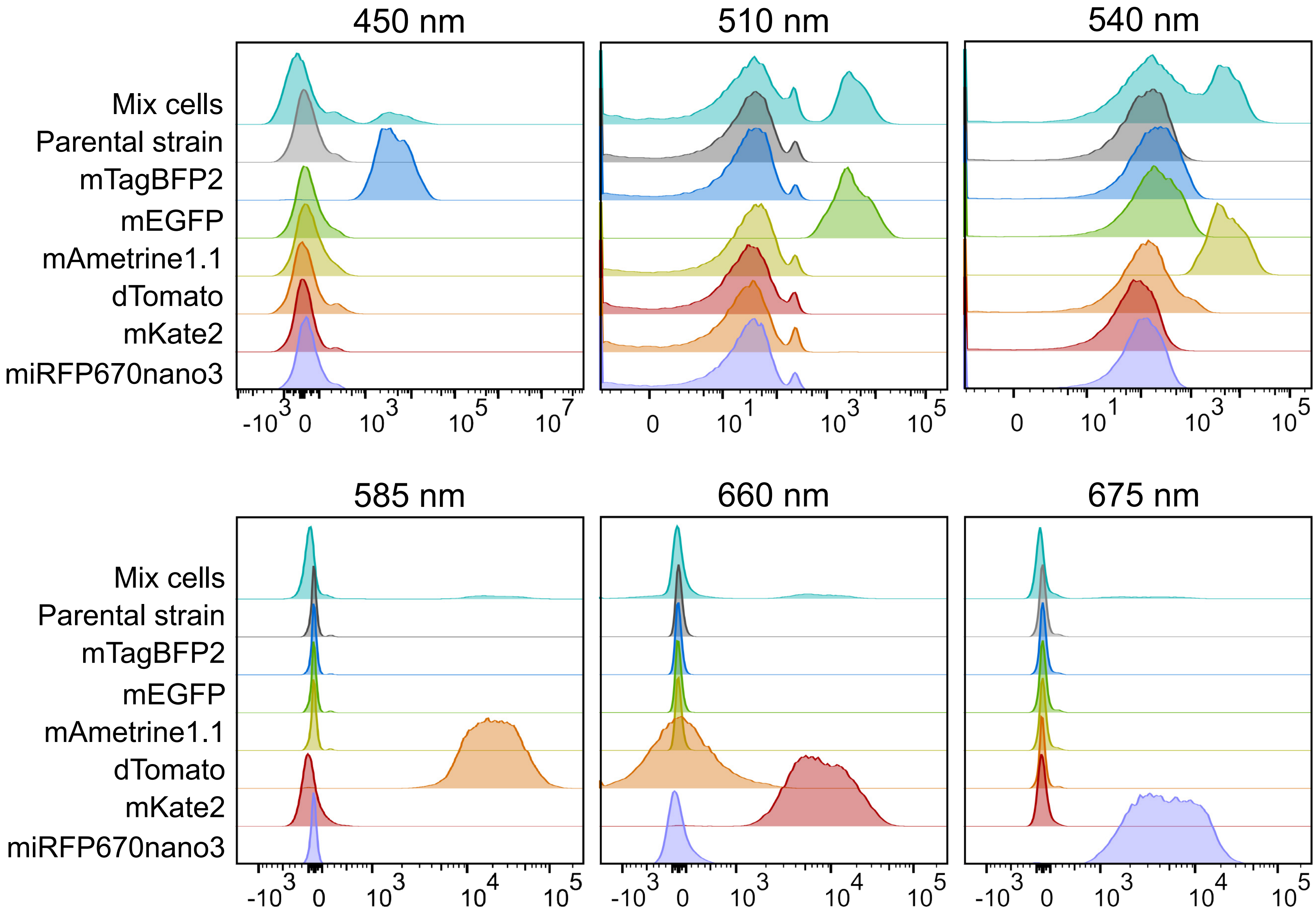

Figure S4. Representative flow cytometry profiles of an *in vivo*-derived CandiChrome sample.
Fluorescence intensity distributions in the indicated emission channels for a mixed-cell sample recovered from day 1, Comp5 of the *in vivo* competition assay. Individual CandiChrome strains and the parental strain (SC5314) are shown for comparison.

Table S1. Strains used in this study.

Strains	Genotype	Source
CAY12597	SC5314 (Wild-type)	[1]
CAY16975	SC5314 Eno1-mTagBFP2 fusion	This study
CAY17099	SC5314 Eno1- mTurquoise2 fusion	This study
CAY16965	SC5314 Eno1- mStayGold fusion	This study
CAY16971	SC5314 Eno1- mNeonGreen fusion	This study
CAY17002	SC5314 Eno1- mEGFP fusion	This study
CAY17060	SC5314 Eno1- mAmetrine1.1 fusion	This study
CAY17323	SC5314 Eno1- dTomato fusion	This study
CAY16999	SC5314 Eno1- mScarlet-I fusion	This study
CAY16902	SC5314 Eno1- mScarlet3-S2 fusion	This study
CAY17058	SC5314 Eno1- mKate2 fusion	This study
CAY17299	SC5314 Eno1- miRFP670nano3 fusion	This study
CAY17295	SC5314 Eno1- BDFP1.6 fusion	This study
CAY17302	SC5314 Eno1- smURFP fusion	This study
CAY17802	SC5314 pENO1- mTagBFP2 Neut5L	This study
CAY18103	SC5314 pENO1- mTurquoise2 Neut5L	This study
CAY18111	SC5314 pENO1- mStayGold Neut5L	This study
CAY17804	SC5314 pENO1- mNeonGreen Neut5L	This study
CAY18129	SC5314 pENO1- mEGFP Neut5L	This study
CAY18106	SC5314 pENO1- mAmetrine1.1 Neut5L	This study
CAY18109	SC5314 pENO1- dTomato Neut5L	This study
CAY18127	SC5314 pENO1- mScarlet-I Neut5L	This study
CAY18131	SC5314 pENO1- mScarlet3-S2 Neut5L	This study
CAY18114	SC5314 pENO1- mKate2 Neut5L	This study
CAY18116	SC5314 pENO1- miRFP670nano3 Neut5L	This study
CAY18126	SC5314 pENO1- BDFP1.6 Neut5L	This study
CAY18321	SC5314 pENO1- smURFP Neut5L	This study
CAY19263	SC5314 Cox4-dTomato; Vph1-miRFP670nano3; Efg1-mTagBFP2;Ras1-mNeonGreen	This study
CAY18410	SC5314 pENO1-mEGFP+ pENO1-miRFP670nano3 Neut5L	This study
CAY18333	SC5314 pENO1-mEGFP+ pENO1-mTagBFP2 Neut5L	This study
CAY18336	SC5314 pENO1-mEGFP+ pENO1-dTomato Neut5L	This study

CAY18335	SC5314 pENO1-mEGFP+ pENO1-mKate2 Neut5L	This study
CAY18334	SC5314 pENO1-mEGFP+ pENO1-mAmetrine1.1 Neut5L	This study
CAY18407	SC5314 pENO1-mTagBFP2+ pENO1-mAmetrine1.1 Neut5L	This study
CAY18342	SC5314 pENO1-mTagBFP2+ pENO1-mKate2 Neut5L	This study
CAY18344	SC5314 pENO1-mTagBFP2+ pENO1-miRFP670nano3 Neut5L	This study
CAY18405	SC5314 pENO1-mTagBFP2+ pENO1-dTomato Neut5L	This study
CAY18352	SC5314 pENO1-mAmetrine1.1 + pENO1-mKate2 Neut5L	This study
CAY18350	SC5314 pENO1-mAmetrine1.1 + pENO1-miRFP670nano3 Neut5L	This study
CAY18341	SC5314 pENO1-mAmetrine1.1 + pENO1-dTomato Neut5L	This study
CAY18404	SC5314 pENO1-dTomato + pENO1-mKate2 Neut5L	This study
CAY18409	SC5314 pENO1-dTomato + pENO1-miRFP670nano3 Neut5L	This study
CAY18338	SC5314 pENO1-miRFP670nano3 + pENO1-mKate2 Neut5L	This study

1. **Bennett RJ, Johnson AD.** 2006. The role of nutrient regulation and the Gpa2 protein in the mating pheromone response of *C. albicans*. Mol Microbiol **62:** 100-119.

Table S2. Oligonucleotides used in this study.

Number	Sequence (5’->3’)
6060	GGACCGCCGCGGTAAACAAGTGGTATTCAAGCACAAT
6061	GGACCGGAGCTCCAGGAAGGACGATGAAGGA
4568	GGACCGCTCGAGCGGATCCCCGGGTTAATT<br>AACGGTATGGTTTCAAAAGGTGAAGAAGTT<br>ATTAAAG
4569	GGACCGCTCGAGTTATTTATACAATTCATC<br>CATACCATACAAAAACAA
4438	CTCAACCATAGCAATCATGG
9795	GGACCGGGTCTCTCAGTCATTTGTATCTTTAGTAGACATGATTGT
9728	GGACCGGGTCTCTCATTGTTGTAATATTCCTGAATTATCAAT
9799	GGACCGGGTCTCTTAAAGTAAAACCAGACTTTGATTTGATT
9800	GGACCGGGTCTCTAGTATGGTAATAGGAAGTCAAAAGAAAGA
9880	GGACCGGGTCTCTAATGGTTTCAAAAGGTGAAGAAGTTATTAAAG
9881	GGACCGGGTCTCTTTTATTTATACAATTCATCCATACCATACAAAAAC
9882	GGACCGGGTCTCTAATGGGTGGTAGTGGTATGGTTTC
9883	GGACCGGGTCTCTTTTAGTTCAATTTATGACCTAATTTTGATGG
9884	GGACCGGGTCTCTTTTATTTATACAATTCATCCATACCCATAACA
10055	GGACCGGGTCTCTTTTATGACATAGCTTTAATAATATAATCAAAATATGGA<br>G
10047	GGACCGGGTCTCTTTTACTATCTATGACCTAATTTTGATGGTAAATCA
10054	GGACCGGGTCTCTTTTAAATTTCAGTTTCTGAAATAACTCTAGC
10049	GGACCGGGTCTCTTTTATGATTGTTGAATAGCAATACCCA
10053	GGACCGGGTCTCTTTTATTATAAATGAGCTTCTAAAGTTTCAGATTG
10048	GGACCGGGTCTCTTTTACTATTTATATAATTCATCCATACCTGGAGTA
10050	GGACCGGGTCTCTTTTACTATTTATATAATTCATCCATACCTAAAGTAATA<br>CCA
10056	GGACCGGGTCTCTTTTACTATGAACCACCTGAACCAC
10051	GGACCGGGTCTCTTTTACTAATATAATTCATCCATACCACCTG
10052	GGACCGGGTCTCTTTTATTATTTATATAATTCATCCATACCTAAAGTAATA<br>CCA
9547	ATCTTGAGAATCGAAGAAGAATTAGGTTCTGAAGCTATCTACGCTGGTAA<br>AGATTTCCAAAAGGCTTCTCAATTGGGTGGTAGTGGTATGGTTTCTAAAG
9548	TTTGACTGCAGCTCAGTGATTAAGAGTAAAGATGGGTAAAAAATTATCAT<br>TTAATTAGTTCATATATTCAAGATGGCGGCCGCTCTAGAACTAGTGGATC

9739	AGAATCGAAGAAGAATTAGGTTCTGAAGCTATCTACGCTGGTAAAGATTT CCAAAAGGCTTCTCAATTGGGTGGTAGTGGTATGGTTTCAAAAGGTGAAG
7764	GTGATGATTTGACTGTCACTAACC
9319	GTGACTCCATCACCCAGTTT
7763	GATGCTTGGGTCCACTTCT
4906	ACTTTAATCCTTAGTCTTCTATTCTGAGAT
7832	ACGCAATTAATGTGAGTTAGC
4439	GCGAAAAAGTGGGCACTAAG
4907	CAAATTCATTGGAGCGATATCG
10243	AAAGTCTCTAGATGCTGGCAATGTGGTACTGTCTTGAAGGCCAAATACTT GGGTGAACCAGGAATGGCTCATCATGGTGGTAGTGGTATGGTTTCAAAAG
10244	TAGTAGAGTTCGCCAAAAGAGAACCATGAAATATATATAAAAAATAGAA TATAGCAATTGTTGGGGGTCTGTCGGGCGGCCGCTCTAGAACTAGTGGAT C
10245	CTTTGATTGGTCCTGGTGCT
5222	CTTCACCTTCAATTTCAAATTCATGAC
10246	ATGTCGAAATATTTTGAAGGTGGTGGTTCTGCTTTTGAACCATTTACTTTT AAAGGTTTATTAGACAGTGTTTTAGGTGGTAGTGGTATGGTTTCTAAAG
10247	ATGTTTAATGTTTAATGTTTATTATATATATTCAAATAAATATAATTCTTAA TTCTTTTTTATAAATTAATTGAGGCGGCCGCTCTAGAACTAGTGGATC
10248	TAATGCATTTGGTCCCACCG
4360	GTGGTTTCAGTGGCTACAAC
4446	CAAGGTTCAGTTCACCCTTCACCCCAACAACATCAAGCTAATCAATCAGC TAGCACTGTTGCCAAAGAAGAAAAGGGTGGTAGTGGTATGGTTTCTAAAG
8774	CTTTCCAATCATTTGTTAATGAAATATATGCTATAATCTAATTTGGAATTT ATGGCAGAAAGCAGAAGGTGATGTACACCGGCCGCTCTAGAACTAGT
5021	AGCGGTAATGGGAACAGTATA
5088	GGACCGGGTACCGCATAATCTCTTTAAACT
5089	ATTATCTTCTTCACCTTTAGAAACCATGGT
5090	ATGGTTTCTAAAGGTGAAGAAGATAAT
5091	TTTATACAATTCATCCATACCCATAACATCA
5092	TGATGTTATGGGTATGGATGAATTGTATAA
5115	GGACCGCTCGAGGTTAGTTTAAGATTACAA
5101	TTGTCTTGGGAATGTAAATTTAAATGAAT
